# Quantitative molecular detection of larval Atlantic herring (*Clupea harengus*) in stomach contents of Atlantic mackerel (*Scomber scombrus*) marks regions of predation pressure

**DOI:** 10.1038/s41598-021-84545-7

**Published:** 2021-03-03

**Authors:** Bridie Jean Marie Allan, Jessica Louise Ray, Maik Tiedemann, Valeriya Komyakova, Frode Vikebø, Katrine Sandnes Skaar, Martina H. Stiasny, Arild Folkvord, Richard D. M. Nash, Erling Kåre Stenevik, Olav Sigurd Kjesbu

**Affiliations:** 1 Institute for Marine Research, 5817 Bergen, Norway; 2 Department of Marine Science, University of Otago, Dunedin, 9016 New Zealand; 3 NORCE Norwegian Research Centre AS, NORCE Environment, 5008 Bergen, Norway; 4 Institute for Marine and Antarctic Studies, University of Tasmania, Hobart, TAS 7001 Australia; 5 University of Bergen, 5020 Bergen, Norway; 6 Centre for Environment, Fisheries and Aquaculture Science (Cefas), Lowestoft, NR33 0HT Suffolk UK; 7 Present Address: Department of Marine Science, University of Otago, Dunedin, 9016 New Zealand

**Keywords:** Molecular biology, Marine biology

## Abstract

Mortality rates in the early life-history stages of fishes are generally high yet identifying the causes remain unclear. Faltering recruitment rates of Atlantic herring (*Clupea harengus*) in the Norwegian Sea indicate a need to identify which mortality factors influence larval herring survival. Previous research suggests that increased predation pressure by Atlantic mackerel (*Scomber scombrus)* may contribute to the disconnect between spawning stock biomass and recruitment. To quantify the contribution of predation pressure by Atlantic mackerel to herring larval mortality, two research cruises were conducted within a probable “hot spot” (67–72° N) for intensified mackerel predation based on particle drift simulations. Mackerel stomach contents were analysed for herring larvae content using droplet digital polymerase chain reaction (ddPCR) with a quantitative molecular detection assay specific for herring. The ddPCR results demonstrate clear predation by mackerel on herring larvae and also suggest that the alternative use of visual examination may give misleading results. Our results show that mackerel should be considered a potentially important predator on herring larvae. The quantitative molecular assay presented here shows great promise as an efficient and specific tool to correctly identify and quantify predation pressure on early life-history stages of fishes.

## Introduction

The survival during early life-history stages (ELHS) of fishes are critical for the replenishment and abundance of fish species in marine ecosystems^1^. As such, any changes during ELHS can translate into large scale ecological changes with the potential to erode the capacity of populations to resist and recover from perturbation. Annual cohort strength is underpinned by rapid growth and successful predator evasion during the larval period^2,3^. Mortality during early ontogeny is generally high and represents a strong selective force, limiting recruitment success for many fish species^2,4^. Hence, starvation and predation pressure can exert significant regulatory control on larval fish affecting recruitment into the juvenile and subsequent adult populations, with mortality rates often exceeding 95%^5–7^. However, quantifying the true impact of predation on larval fishes is difficult owing to the patchy distribution of both predators and prey.

Predation on pelagic fish larvae is a difficult process to document^2^, though jellyfish and planktivorous fish have been suggested to be the main predators^2,8–10^. Most predation studies to date have estimated predation by morphological analysis of predator stomach contents including identification of digested prey items based on exoskeletons and calcified structures such as otoliths^10,11^. However, larval fish are small, soft bodied, translucent organisms and are often macerated and digested beyond recognition, making predation difficult to quantify^12–14^. Further, the inability to correctly identify prey items owing to the level of taxonomic resolution one can achieve through visual analysis alone makes the comparison across studies difficult. This problem reduces the ability for hypothesis-driven ecosystem modelling for predicting the impact of shifts in predator fields due to climate fluctuations and change.

Moving beyond morphological analyses, molecular tools have been developed as a way to circumvent some of the limitations of visual identification of stomach contents^15^, see^16^. Molecular analyses of fish stomach contents can identify a molecular signature > 24 h after ingestion, although the length of this detection ability being negatively affected by stomach temperature during digestion^17^. By eliminating the requirement for visually identifiable morphological features in partially digested stomach content samples, molecular analysis of predator stomach content might be a highly effective tool to assess predator–prey interactions in a variety of predator types^16–20^. Through the use of taxon-specific primers, the magnitude of predation on individual species present in mixed communities can be quantitatively assessed^16,19^.

Forming an ecologically and commercially important component of the Northeast Atlantic ecosystem, Norwegian spring-spawning herring (NSSH) is one of the largest fish stocks in the world. The adults spawn between February and April along the Norwegian coast between 58° and 69° N, where the eggs adhere to the substratum, and hatching occurs approximately 3 weeks after spawning^21^. After hatching, NSSH larvae are transported northwards by the Norwegian Coastal Current (NCC) to the nursery areas in fjords along the coast or into the Barents Sea^22^. Herring larvae are generally little affected by sporadic restricted feeding opportunities in experimental settings, indicating that predation rather than starvation is the main mortality regulator in the natural environment at that stage in life^23^. Generally, NSSH spawning stock biomass (SSB) and the subsequent level of recruitment is positively related^24^. However, despite a reasonably large SSB producing high numbers of larvae, no strong year classes were produced between 2004 and 2015^25^. The causes of the recently seen lack of a large year class remains unclear, despite^10,26^ suggesting that spatiotemporal overlap with high food concentrations and predators are likely to be important regulators in Norwegian coastal waters for the survival of herring larvae, as well as large-scale, long-term climate oscillations^27^.

For the Norwegian Sea, the expansion of Atlantic mackerel into Norwegian waters^28,29^ has coincided with the decrease in larval NSSH successfully recruiting into the juvenile population^30^. As such, the advance of mackerel may have direct implications on larval NSSH survival during the migration north^10^. However, quantifying the full impact of predation by mackerel is challenging owing to limitations in correctly identifying consumed fish larvae. It is a key challenge to understand the mechanisms underlying the high variability in larval mortality for managing this valuable marine resource and key ecosystem component. This issue is especially important in management strategy evaluations (MSE) (see^31^ and references therein). Generally, the ability to predict future standing stock biomass is dependent on in-depth knowledge about mechanisms underlying recruitment success (e.g.^32^. Therefore, recruitment success can largely define the precautionary level of applied fishing mortality.

In this study, PCR primers specific for herring (Pacific herring, *Clupea pallasii*, and Atlantic herring, *C. harengus*)^33^ were validated and implemented for quantitative detection of herring larvae in the stomach content of Atlantic mackerel. In line with previous DNA-based barcoding studies of larval fish predation^34,35^, this assay targets the mitochondrial 16S ribosomal RNA gene (mt16S), a high copy number DNA target^36^. To verify the detection ability and specificity of the developed assay as a tool to quantify predation on herring larvae, the assay was tested on mackerel stomachs collected from between 68 and 72° N across the Norwegian coastal shelf edge based on preceding considerations of likely spatiotemporal overlaps. The objectives of this novel study were (1) to evaluate the species specificity and sensitivity of the herring-specific PCR primers, (2) to quantify the degree to which herring larvae may be found in Atlantic mackerel stomachs, when precisely measured as number of gene copies, and (3) since the only herring larvae occurring in this area at this time are NSSH, to consider whether such top-down control may impact subsequent NSSH cohort survival. Hence, the development of a more robust and importantly, a more comparable method of estimating predation in the field may allow for more accurate predictions of recruitment success of the fish stock of interest given the spatiotemporal overlap of its ELHS and the foreseen key predator(s).

## Material and methods

### Sampling strategy and plan

**Figure 1:**
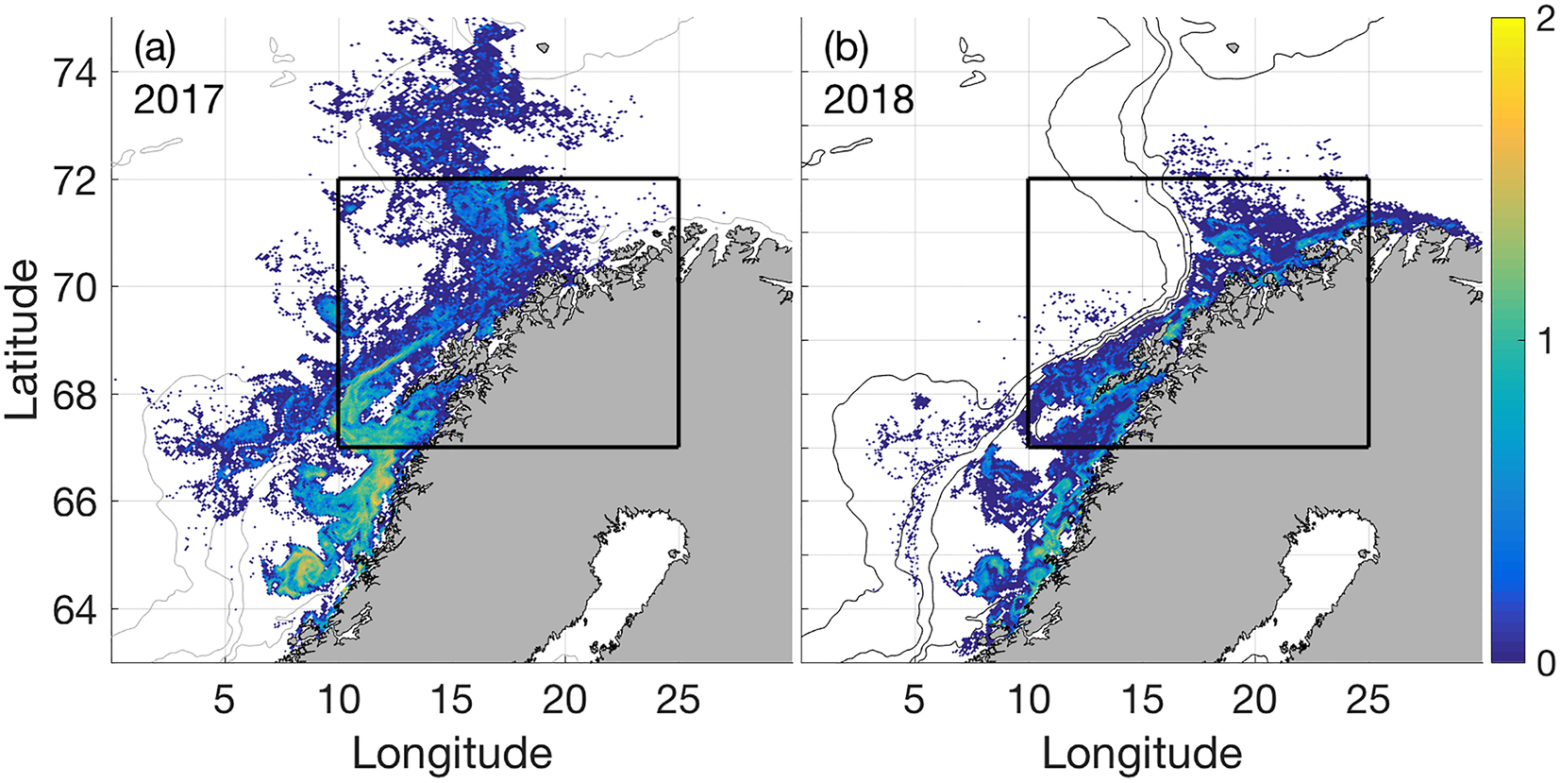
Modelled NSS herring larvae distribution, (abundance = log n) (**a**) 2017 and (**b**) 2018 based on an individual-based particle drift model initiating particles at the time of hatching between March 15th until April 20th at well-known spawning grounds but mainly at Møre (about 63° N).

**Figure 2:**
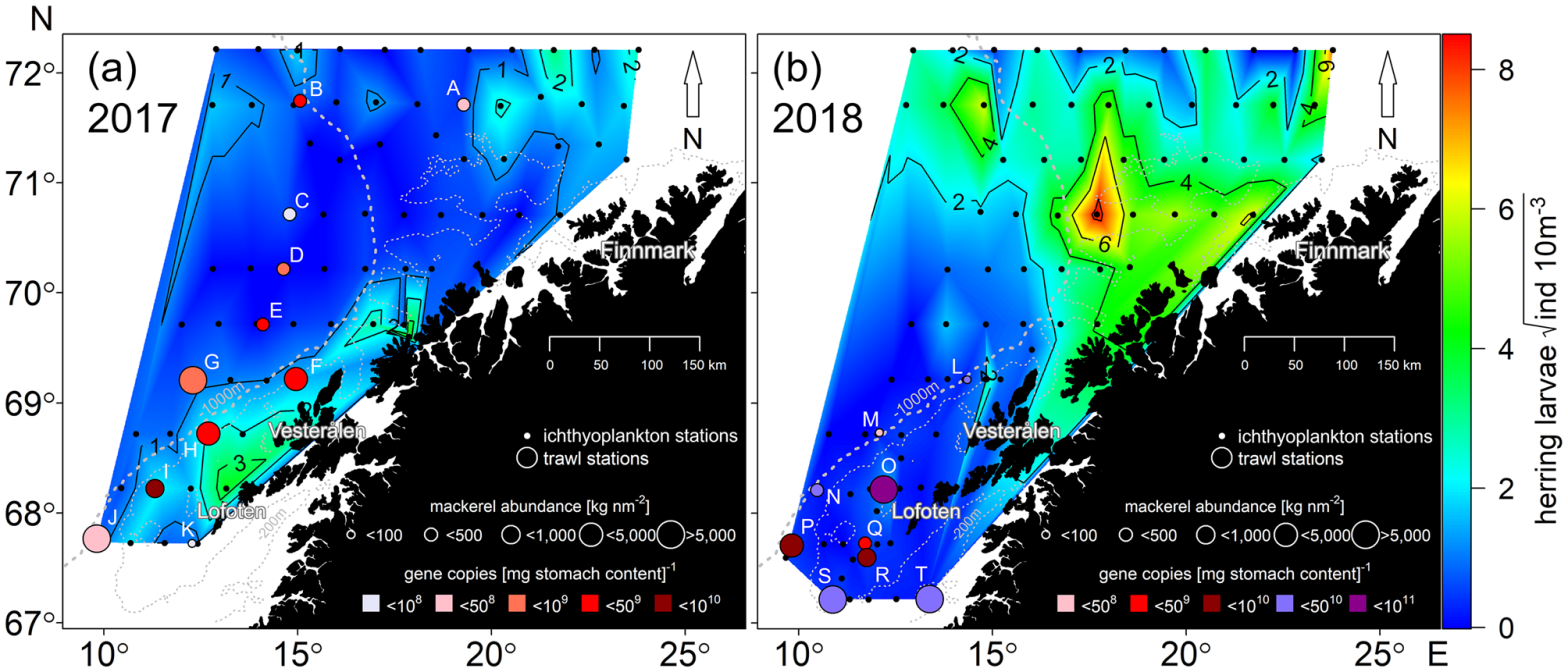
Collated information from the IMR larval Norwegian spring-spawning herring (NSSH) research cruise in (**a**) 2017 and (**b**) 2018. The interpolated area indicates NSSH larval densities, black (small) circles depict plankton sampling stations, coloured circles depict trawl stations of mackerel, where mackerel biomass densities are represented by circle size, whereas colours of these circles depict mt16S gene copies of NSSH in mackerel stomachs. Sampling stations are consecutively labelled (A-T), where NSSH larvae were genetically observed in mackerel stomachs (Table 2). For visualization purposes, larval densities are given as the square root of n.

The cruise planning was set up to identify the most suitable sampling area and to detail a proper sampling strategy to address hypotheses related to mackerel predation on herring larvae: Atlantic mackerel migrate into Norwegian coastal waters between May and July^29,37^ with potential spatiotemporal overlap with drifting NSSH larvae^38^. Time series information from historic “NSSH Postlarvae Surveys” of the Institute of Marine Research (IMR) (e.g.^39^ as well as model outputs (Fig. 1a,b) from an individual-based particle-tracking model (IBM) were used to inform the spatiotemporal sampling design (Fig. 2a,b). The IBM was forced by daily mean 3D currents from an ocean model archive^40^, and run with the ROMS (Regional Ocean Modelling System) model^41^ on a 4 × 4 km horizontal grid with 32 vertical sigma layers. A total of 198,580 particles were released at well-known spawning grounds at Møre, near the Haltenbanken, Sklinna and Røst (50, 20, 10, 20% respectively; see^38,42^, i.e. from about 63° to 68° N, respectively, with a Gaussian-shaped hatching intensity between March 15th and April 20th. Particles resembling drifting larvae undergo a diel migration between 5 and 40 m—shallow during night and deeper during day—by swimming at a speed of 0.1 body lengths per second. Larvae are initiated (hatch) at 9 mm and grow 0.4 mm per day^43^. The outputs provided corresponding expected distribution of NSSH larvae along the coast. Following these results (see “Result” Section), the research cruise was scheduled to take place around mid-June, more specifically on 9–27 June 2017 and 5–25 June 2018, using in both cases the IMR R/V *Johan Hjort*. The cruise direction was from north to south in order to identify the boundaries of northward drifting larvae as well as adult mackerel. More detailed sampling procedures are described in the following sections.

### Herring larvae sample collection

NSSH larvae were collected in a predefined sampling path with west–east transects (30 NM apart) and successive stations (20 NM apart). Samples were collected using either an ICES (International Council for the Exploration of the Sea) standard 2 m Midwater Ring trawl (MIK) (main net mesh size: 1600 µm, cod end mesh size: 500 µm)^44^ or a macrozooplankton trawl (cod end mesh size: 3 mm stretched mesh); the sampled NSSH larvae during the historic surveys were generally > 10 mm in standard length^38^. Double oblique hauls down to maximum 100 m or 5–10 m above the sea floor were conducted using a vessel speed of 2–3 knots (1–1.5 m s^−1^). The cod end was washed down at the end of each haul. Filtered water volume was calculated based on mechanical flowmeter readings (General Oceanics, model 2030R, https://www.generaloceanics.com/mechanical-flowmeters/) before and after each haul. NSSH larvae were sorted and counted to estimate densities [individuals per 10 m^3^].

### Mackerel sample collection

Mackerel were captured using pelagic trawls, either a Multpelt 832 trawl (vertical height 35 m) in 2017 or Åkra trawl (vertical height 30 m) in 2018. The trawling speed was ~ 4–5 knots (2.1–2.6 m s^−1^) for ~ 30 min for efficient sampling. All specimens were immediately worked up according to standard procedures onboard, in this case weighing the whole body (fish) weight (in g) and the excised stomach (in g) and staging stomach fullness^45^. No specific permissions for sampling were required as all mackerel were obtained in accordance with Norwegian law concerning animals. Mackerel densities were calculated as swept area estimates from the trawl hauls using the StoX software^46^, which is an open source software routinely used in fisheries for both acoustic and swept area estimates^28^.

### Dissection of mackerel stomachs

Immediately after each net haul, up to ten randomly selected mackerel, all dead when landed onboard, were measured and their intact stomachs excised and immediately stored in 70% ethanol in individual 200 mL polypropylene bottles at − 20 °C until laboratory analysis. Stomach contents were collected by manual dissection 2–4 months after freezing, wet weights recorded and homogenized in an approximate 1:3 ratio of 1 volume of stomach contents to three volumes of autoclaved 1× phosphate buffered saline (pH 8). Samples collected in 2017 were homogenized using a Potter–Elvehjem piston-type Teflon tissue grinder (https://www.kisker-biotech.com/frontoffice/product?produitId=0A-67-08) with radial serrations, while 2018 samples were homogenized in sterile 50 mL polypropylene tubes using a Tissue Ruptor II (QIAGEN, Hilden, Germany), always with a new probe for each sample (i.e. contents from one stomach). To test for historic *C. harengus* DNA in apparently empty stomachs (2018: N = 6), these samples were placed in sterile 50 mL tubes with 10 mL 1× PBS buffer and vortexed vigorously for 10 s to release residual prey tissue/DNA from the stomach lining. The weight of tissue in empty stomachs was artificially set to 0.01 g in order to be able to calculate ddPCR gene copies per g stomach content. Subsamples of these mock “homogenates” were taken and processed together with the remaining samples. To account for homogenization efficiency and stomach content patchiness, we took five replicate subsamples (approx. 0.2 g wet weight) from each stomach homogenate into individual 1.5 ml microcentrifuge tubes, noting the wet weight of each replicate. Wet weights were later corrected to remove the weight of the added PBS buffer prior to homogenization. Homogenates and subsamples were stored at − 20 °C until DNA extraction. To ensure the homogenization process did not introduce contamination, we collected one blank subsample of sterile PBS buffer in which a new homogenization probe had been inserted and pulsed, and one blank subsample of sterile PBS buffer alone. These blanks were treated as samples and processed accordingly for DNA extraction and ddPCR analysis (see below).

### Visual analysis of mackerel stomach contents

A subset of the preserved mackerel stomachs (2017: N = 96) were thawed and inspected under a stereomicroscope for recognition of herring larvae, searching also for any presence of their otoliths, i.e., objects considered especially robust to degradation^47^.

### Genetic analyses of NSSH larvae in mackerel stomachs

#### DNA extraction

Twenty-four hours prior to DNA extraction, cellular material present in subsamples was lysed with 200 µL Buffer ATL (QIAGEN) and 20 µl (20 mg ml^−1^) Proteinase K (QIAGEN) in a 56 °C heating block for approx. 16 h. DNA in lysates was purified on a QIAsymphony SP automated platform running software version 4.0 and using the DSP DNA Mini Kit (QIAGEN, cat.no: 937236) with the Tissue_LC_200_V7_DSP protocol. Elution volume used was 100–200 µl and samples were collected in a 96-well elution microtube plate (QIAGEN). Purified DNA was stored short-term at 4 °C and long-term at − 20 °C. For specificity testing (see below), tissue samples from fish, zooplankton and phytoplankton (Table 1) were subjected to DNA extraction using the DNeasy Blood & Tissue mini kit (QIAGEN) according to manufacturer instructions.

**Table 1:** Genomic DNA used to confirm specificity of *C. harengus* ddPCR assay.

Organism	Group	Tissue type	ddPCR result
*Clupea harengus*	Fish	Muscle	**+**
		Whole Larvae	**+**
*Scomber scombrus*	Fish	Muscle	**−**
Gadoid spp.	Fish	Entire juvenile	**−**
Pleuronectiformes spp.	Fish	Entire juvenile	**−**
*Mallotus villosus*	Fish	Muscle	**−**
		Whole Larvae	**−**
*Gadus* spp.	Fish	Muscle	**−**
*Oncorhynchus mykiss*	Fish	Muscle	**−**
*Salmo salar*	Fish	Muscle	**−**
*Calanus* spp.	Copepod	Whole animal	**−**
*Oikopleura dioica*	Tunicate	Whole animal	**−**
*Skeletonema* spp.	Phytoplankton	Cells	**−**
*Phaeocystis pouchetti*	Phytoplankton	Cells	**−**

#### Molecular detection assay

A literature search for quantitative PCR assays targeted to *Clupea harengus* resulted in one study that focused on quantification of a 69 bp fragment of the mitochondrial small subunit ribosomal RNA gene (mt16S) from Pacific herring (*Clupea pallasii*) in faecal DNA of predator organisms^33^. Comparison of the *C. pallasii*-specific primer sequences (forward 5′-CGCCCACCAATCACGAA-3′ and reverse 5′-ACGTTTGTGCCAGTATCACGTT-3′) with the mitochondrial genome sequence from *C. harengus* (GenBank accession AP009133.1) revealed sequence similarity, suggesting that the *C. pallasii*-specific primers might be used to quantify *C. harengus*. The fact that populations genetically related to *C. pallasii* appear in some fjords in northern Norway^48,49^ was ignored here as any spatiotemporal overlap between the two species’ larvae is extremely unlikely due to the drift routes of the NSSH larvae^50^ and the current off-shore sampling program (Fig. 2).

#### Assay optimization and specificity

All droplet digital PCR (ddPCR) analyses were performed using a QX200 system with QX200 EvaGreen Supermix (Bio-Rad, Hercules, CA, USA). It was necessary to modify assay conditions as described in Bowles et al.^33^ for the ddPCR platform. Twenty-two microlitre reactions containing 1× supermix, 2.2 pmol (100 nM final concentration) of each primer and ultrapure water (q.s. 16.5 µl) were prepared inside a laminar flow safety cabinet inside a template-free pre-PCR area. Reaction mixes were then transferred to the main laboratory where 5.5 µl of template was added (final volume 22 µl). Twenty-microlitres from each PCR reaction were then transferred to sample wells in a droplet generation cartridge (Bio-Rad). Oil wells in the same cartridge were filled with 70 µl of droplet oil for EvaGreen detection. Emulsions of PCR reactions were then prepared using a droplet generator (Bio-Rad), and 40 µl of each resulting emulsion was transferred to a sterile PCR plate. Once all emulsion reactions had been transferred, the plate was sealed with pierceable aluminum foil (Bio-Rad). Amplification was conducted in a C1000 Touch thermocycler with deep-well module (Bio-Rad) with the following program: 95 °C for 5 min; 40 cycles of 95 °C for 30 s, 60 °C for 60 s; 4 °C for 5 min; 90 °C for 10 min, 4 °C infinite hold. All cycling steps were conducted with a 2.5 °C/s ramp rate. Plates not immediately read on the droplet reader were stored at 4 °C in the dark overnight prior to reading the following day. PCR plates were equilibrated to room temperature for 10–15 min on the laboratory bench prior to droplet reading. Droplet reading was conducted on the DX200 Droplet Reader (Bio-Rad) according to manufacturer instructions.

Assay specificity was determined by testing the assay on genomic DNA from *C. harengus* muscle tissue and whole larvae in addition to DNA extracted from a range of fish tissue, zooplankton and phytoplankton samples (Table 1). To identify best template dilutions for ddPCR, single subsamples from two random fish sampled at each station were selected for testing the effect of template dilution on ddPCR efficiency. Template DNA was diluted in 10 mM Tris–Cl, pH 8.0, then tests including undiluted, 1:10 and 1:100 dilutions of samples were used for ddPCR analysis. The following dilutions were presently found appropriate to overcome sample saturation: 1:10, 1:40 and 1:100. To ensure no cross-contamination during sampling and DNA extraction, blank samples were processed together with stomach samples. All ddPCR runs included at least one no template control (NTC), one positive control (genomic DNA from *C. harengus*), one negative control (genomic DNA from *S. scombrus*).

#### Quantitative sample analysis

The ddPCR quantification of *C. harengus* mt16S gene copies (g stomach content)^−1^ (referred to hereafter as mt16S gene copies) was performed on individual samples using appropriate dilutions of template DNA (see above). Reactions yielding less than 13,000 or more than 21,000 droplets were repeated to improve reaction preparation (theoretical ideal 20,000 droplets/reaction). Samples yielding “No Call” (i.e. no positive droplets) were either repeated to verify negative results or repeated with higher template dilutions to overcome sample saturation. Raw data showing the number of positive events (droplets) per µl in ddPCR reactions were normalized to mt16S gene copies per sample.

### Statistical analysis and production of maps and graphs

A number of five replicate subsamples were taken from each stomach homogenate and averaged. The 2017 and 2018 data sets were analysed separately and the presence of any outliers tested (see statistical packages below) but with negative results, so no gene copy values were omitted. A one-way analysis of variance (ANOVA) was used to compare the differences in these numbers of mt16S gene copies between different sampling stations. Tukey’s HSD (honestly significant difference) post hoc tests were used to examine the differences detected by the ANOVA. Normality and homoscedasticity of residuals of the models were verified by residual-fit plots. The raw data violated the assumption of normality; therefore, a log-transformation was applied to improve the distribution of the data. The model was run in the package aov and Tukey HSD in R^51^, as implemented in RStudio v.1.1.423^52^. Statistical relationships between the number of mt16S gene and predator parameters such as fish weight and weight of stomach content were undertaken using a Pearson’s type correlation. Additional packages maps^53^, mapdata^54^, marmap^55^, akima^56^, GISTools^57^, ggplot2^58^, reshape2^59^, grid^51^ and gridExtra^60^ were employed for data handling and visualization.

## Results

### Simulated herring larval distribution

According to the particle drift model, high NSSH larval densities would be expected to appear in June 2017 along a long stretch of the Norwegian coast, i.e. from about 64° to 69° N (Fig. 1a). As noticed, larvae are clearly drifting in the NCC but also displaying lower concentration patches “off-track” due to eddy shedding. In contrast, high concentrations of herring larvae occur in numerous patches in 2018 along the coast, likely due to more south-westerly winds resulting in Ekman transport towards the coast and concurrent downwelling (Fig. 1b.). Patches are found all the way to the Finnmark coast (see geographical annotation in Fig. 2), i.e. from 64° to 71° N. Also, noticeably, the dispersal in 2017 is towards the far western parts of the Barents Sea (up to 74° N), while in 2018 it is towards the south-eastern parts of the Barents Sea (72° N).

### Observed herring larval distribution

NSSH larvae occurred in patches during both surveys, in 2017 very close to the coast off the Lofoten peninsula (68°–69° N; ≈ 10 larvae 10 m^−3^) (Fig. 2a) and in 2018, primarily in one large patch north of Vesterålen (≈ 71° N; > 50 larvae 10 m^−3^) but generally with an extensive oceanic distribution reaching to the northern fringe of the survey area (≈ 72 °N) (Fig. 2b) (note rooted larval numbers in both Fig. 2 panels). For 2017, apart from the one area with elevated densities, low larval densities were observed over the whole sampling area (< 5 larvae 10 m^−3^). In 2018, low densities were observed off the Lofoten Peninsula. Overall, larval densities in 2018 were higher and more spatially widespread than in 2017.

### Observed mackerel distribution

During both surveys, mackerel were frequently recorded along the drift route of NSSH larvae (Fig. 2). While in 2017 mackerel occurred throughout the sampling area with highest abundances west of the Lofoten Peninsula, mackerel in 2018 were primarily found south of the Lofoten Peninsula. Where mackerel occurred, the biomass varied considerably ranging between 39 and 13 230 kg NM^−2^ in 2017 and 32 and 11 880 kg NM^−2^ in 2018. The presence of mackerel overlapped with low NSSH larval densities in both years.

### Visual analysis of mackerel stomach contents

**Figure 3:**
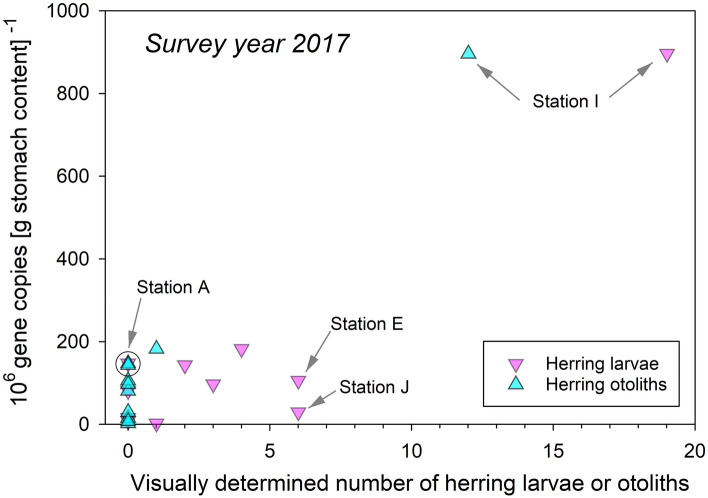
Results of visual examination of mackerel stomachs in June 2017 for presence of herring larvae and otoliths in relation to the corresponding number of mt16S gene copies, split by sampling station. Geographical locations of marked stations are shown in Fig. 2.

Visual examination of the 96 mackerel stomachs collected at 11 stations in 2017 (Fig. 2a) resulted in observations of 41 herring larvae and 13 otoliths. Station I showed an extreme aggregated sum of 19 larvae and 12 otoliths, where 7 larvae and 8 otoliths were detected in a single stomach, and yielded the highest observations of larval remains from stomach contents (Fig. 3). The bulk of larvae and otoliths were present in stomachs from the southern part of the survey area, close to the larval patch at the Lofoten area (Fig. 2a). Altogether only one, single larvae were reported from the four northernmost sampling locations, and then in Station C (Fig. 2a).

Although the number of mt16S gene copies and corresponding number of visually reported herring larvae and otoliths (pooled or held apart) appeared positively correlated (*p* < 0.001), these series of statistical relationships were complicated by an exceedingly high leverage point (≥ 0.843) (Station I) (Fig. 3). Notably, four samples showing neither larvae nor otoliths yielded from 8 × 10^6^ (Station K) to 148 × 10^6^ mt16S gene copies (Station A), both locations in the outer part of the study area (for reference: the corresponding maximum for all 11 stations in 2017 being 896 × 10^6^ (Station I)) (Fig. 3). Even more striking, Station J yielded low gene copies, i.e. 30 × 10^6^ mt16S gene copies, yet the remains from 6 herring larvae were observed (Fig. 3). It is possible that the herring larvae that were observed were misidentified, a common issue when identifying stomach contents. Furthermore, Station E yielded 106 × 10^6^ mt16S gene copies, with also six larvae being reported (Fig. 3). Presence of a single herring otolith corresponded with at least ≈ 75 × 10^6^ mt16S gene copies (Fig. 3).

### Molecular detection of NSSH DNA in mackerel stomach contents

In total, from the 11 stations studied in 2017 and the 9 studied in 2018 (Fig. 2), DNA was analysed from homogenized tissue samples collected from 174 mackerel stomachs. Results ranged from below detection limits to 42.16 × 10^9^ mt16S gene copies (2018; Fig. 2b). Hence, an enormous range of detectable DNA signals was observed, with highest rates of detection in samples collected during 2018 (Fig. 2).

ANOVA confirmed significant differences in ddPCR results between sampling stations in both years (Table 2, F_10,73_ = 10.65, *p* < 0.001 (2017); *F*_8,81_ = 3.631, *p* < 0.002 (2018)). However, there was significant variability between stations despite proximity to each other. For example, most stations between 68° and 70° N had the highest number of mt16S gene copies detected. In 2017 Station I stood out with ~ five to six times as many gene copies than the next two stations with the highest number Station F and H (Table 3, Fig. 2a). The number of mt16S gene copies detected in 2018 was generally higher than that in 2017 for most stations. However, the overall pattern observed in 2018 was similar to the pattern observed in 2017 with southern stations having larger numbers. In 2018, stations south of 69° N had the highest number of mt16S gene copies. In particular, more than 3.58 times mt16S gene copies were detected at Station O compared to the next station with the highest number (Station T) (Table 3, Fig. 2b). Additionally, “visually empty” mackerel stomachs also yielded detectable *C. harengus* DNA signal in some subsamples, although the sample range was from below detection limit to 8.96 × 10^8^ mt16S gene copies in 2017.

**Table 2:** One-way ANOVA results exploring the differences in the number of mt16S gene copies [g stomach content]^−1^ between different sampling stations over two separate sampling periods (years: 2017 and 2018).

Year	Factor	df	SS	MS	F	p
2017	Station	10	322	32.2	10.65	**< 0.001**
	Residuals	73	221.4	3.02		
2018	Station	8	176.5	22.059	3.631	**0.001**
	Residuals	81	492.1	6.075		

Significance accepted at *p* < 0.05 and is highlighted in bold.

**Table 3:** Pair-wise Tukey test results exploring the differences in the number of mt16S gene copies [g stomach content]^−1^ between different sampling stations over two separate sampling periods (years: 2017 and 2018).

2017										
Station number	A	B	C	D	E	F	G	H	I	J
B	**0.041**									
C	**0.004**	NS								
D	NS	NS	**0.043**							
E	NS	NS	**0.013**	NS						
F	NS	**0.003**	**0.000**	NS	NS					
G	NS	**0.043**	**0.004**	NS	NS	NS				
H	NS	**0.001**	**0.000**	NS	NS	NS	NS			
I	**0.001**	**0.000**	**0.000**	**0.001**	**0.004**	**0.024**	**0.001**	**0.041**		
J	**0.001**	NS	NS	**0.02**	**0.004**	**0.000**	**0.001**	**0.000**	**0.000**	
K	NS	NS	NS	NS	NS	NS	NS	**0.031**	**0.001**	NS

2018										
Station number	L	M	O	N	P	Q	R	S		
M	NS									
O	NS	**0.014**								
N	NS	NS	NS							
P	NS	NS	NS	NS						
Q	NS	NS	NS	NS	NS					
R	NS	NS	NS	NS	NS	NS				
S	NS	NS	NS	NS	NS	NS	NS			
T	NS	**0.001**	NS	**0.039**	NS	**0.006**	**0.024**	**0.006**		

Only significant *p* values are displayed (< 0.05).

*NS* not significant.

**Figure 4:**
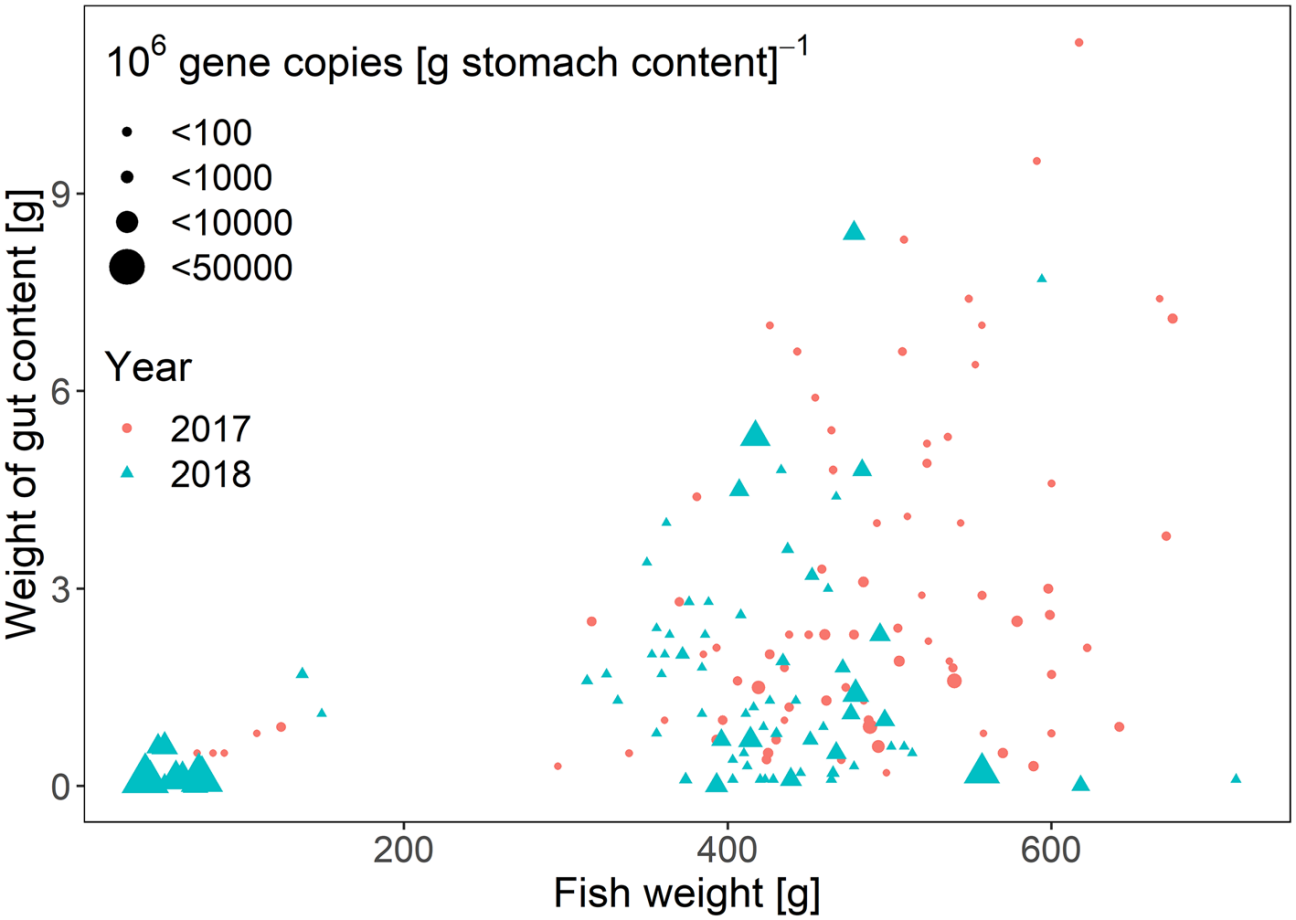
Number of herring mt16S gene copies in mackerel stomachs (size of symbols) in 2017 and 2018 as a function of mackerel whole body (fish) weight and weight of gut content.

We observed no correlation between mt16S gene copies and predator fish weight in 2017 (t = 0.72008 df =_ 82_, p = 0.47), but for 2018 this correlation was strongly negative (t = − 2.6224, df = _88_, p = 0.01) (Fig. 4). There was a negative correlation between weight of stomach content and mt16S gene copies in 2017 (t = − 2.345, df = _82_, p = 0.02), however this was not apparent in 2018 (t = − 1.1571, df = _88_, p = 0.25) (Fig. 4). In general, the variability of mt16S gene copies was high regardless of the predator fish weight and the weight of the stomach content.

## Discussion

Whereas an increasing number of studies have investigated the effects of environmental variability on the recruitment potential of ELHS (e.g.^26,61–63^, the impacts of predation on these vulnerable ontogenetic stages is poorly understood^7^. In our study, we selected the recent mackerel northward expansion and stock size increase^28,29,64^ to investigate resulting predation effects on larvae of Norwegian spring-spawning herring (NSSH), hypothesized to be significant based on the nearly persistent suppression of NSSH recruitment success over more than a decade^25^. As a first approach and to increase our ability to more accurately measure predation pressure, we developed an assay to quantify the number of *C. harengus* mt16S gene copies present in the stomach contents of mackerel collected during the 2017 and 2018 larval drift period. We found that the signal of mt16S gene copies varied significantly both between and within sampling stations, highlighting the apparent randomness or patchiness of mackerel predation on NSSH larvae even within the small geographical area sampled in a trawl haul. Moreover, stomach content weight could not be used as a reliable indicator of predation. For example, mackerel stomachs that appeared to have no visible stomach contents yielded detectable NSSH DNA signals providing strong evidence of past feeding on NSSH larvae. This finding is an important result given that previous attempts to assess predation pressure on fish larvae have used a combination of quantifying the spatiotemporal overlap of interacting species and visually identifying the stomach contents of purported predators^10^. Therefore, the risk of significantly underestimating predation using visual analysis alone is quite high.

The range in normalized DNA results for *C. harengus* detection in mackerel stomach contents across all samples analysed in this study, clearly distinguishes between “high” and “low” predation signals. This enables assessment of predation on *C. harengus* by *S. scombrus* both between fish at each station, as well as between stations. However, it is important to note that this method is not without potential errors of interpretation. Before this or any DNA detection method can be used to more accurately quantify predation, additional information about prey DNA stability and digestion rates after ingestion by predators are required. Therefore, it is not possible to determine where or when the NSSH DNA signal in mackerel stomachs arose, i.e. when each mackerel individual last consumed a NSSH individual. Moreover, a quantitative definition of the relationship between detectable ddPCR signal and ontogenetic stage must be established. Finally, we are unable to discriminate between primary and secondary predation, for example if a predator other than mackerel consumed NSSH larvae and was then consumed by mackerel^65^. The additional DNA degradation that secondary predation events incur, however, makes it unlikely that DNA detection from secondary predation events would overshadow the DNA signal generated from the primary predation event (mackerel directly consuming NSSH larvae) targeted in this study. Despite these limitations, the ability to associate a molecular signal of NSSH larvae with the spatial distribution of mackerel offers a promising opportunity for targeted DNA analyses to contribute to a richer dataset for more accurate quantification of the magnitude of mackerel predation on this important fish resource.

Simulations performed using biophysical coupled models along with observed mackerel trawl data suggest that NSSH larvae overlap with NE Atlantic mackerel in time and space, thus potentially increasing the likelihood of predation. However, whereas contemporary modelling approaches can provide important insights into the potential recruitment dynamics of NSSH larvae, there are limited empirical data on realised predation rates. By quantifying the degree of spatiotemporal overlap between NSSH larvae and mackerel, we attempted to test whether spatiotemporal overlap in predator and prey populations matches with observed molecular predation signal. For 2017, our trawl-based observation of an overlap of adult mackerel with NSSH larvae in the main area of the NSSH drift route^66^ coincided with low larval densities and high mt16S gene copies in mackerel stomach contents, implicating realised predation. In contrast, an absence of mackerel around ≈ 71° N in 2018 corresponded with higher larval NSSH densities, indicating a potential reprieve from predation pressure. The simulated high concentration of NSSH larvae in the Lofoten area in 2017 was in discord with low NSSH abundances in sampling nets, arguing for heightened predation. This is supported by the overall high rates of NSSH DNA detection in mackerel stomachs in the Lofoten area in 2017. Alternatively, the low abundance of NSSH larvae in trawl surveys can also be due to other causes, such as starvation. Herring is, however, generally robust to starvation, especially past the critical window^23^. In summary, our synthesized model, trawl and DNA results suggest that the degree of overlap between mackerel and NSSH larvae may be a reliable predictor of predation pressure, and that quantitative DNA detection may provide an efficient means to validate predictions of predation pressure.

Mackerel begin migrating into Norwegian waters in the spring along a south-north axis and have been observed as far north as the southern coastal waters off Svalbard (≈ 78° N) in July/early August^64^. Based on their sustained swimming speeds (max. sustained swimming speed of 116 cm s^−1^, max. speed 550 cm s^−1^)^67,68^, individuals of this species have the potential to travel northward faster than the Norwegian coastal current (main speed 15–40 cm s^−1^, max. speed 100 cm s^−1^)^50^. Thus, in years in which NSSH larvae hatch late in the season, the potential for overlap is increased^69^. Therefore, it is suggested that enhanced survival of NSSH larvae may occur due to early hatching and early transport of NSSH larvae and larvae towards nursery grounds^38,69^. Since 2007, the largely expanded feeding area of mackerel^29,64^ may thus contribute to a strong top down control of NSSH ELHS in years with high degree of overlap between mackerel and herring larvae. Our observations for 2017 versus 2018 support this expectation.

The results presented here provide evidence that the reliance on spatial overlap data or visually quantifying stomach contents as a tool to quantify predation increases the risk of under- or overestimating predation pressure. As such, we suggest that a combination of methods should be used. The applied quantitative molecular assay shows great promise as a cost-efficient and specific tool to correctly identify and quantify predation pressure on fish populations. Determining the magnitude of predation in migratory animals is challenging owing to the difficulties in predicting the degree of overlap between predators and prey as well as quantitatively measuring predation after such an event has occurred. Analysis of mackerel stomach contents revealed the molecular presence of NSSH larvae. This research is an important step forward in accurately quantifying predation pressure on ecologically and commercially important species.

## Data Availability

Data and statistical code is avaliable upon request.
